# Mid-Term Outcome after Extracorporeal Life Support in Postcardiotomy Cardiogenic Shock: Recovery and Quality of Life

**DOI:** 10.3390/jcm13082254

**Published:** 2024-04-12

**Authors:** Maja Hanuna, German Herz, Andre L. Stanzl, Yupeng Li, Christoph S. Mueller, Christine E. Kamla, Clemens Scherer, Dietmar Wassilowsky, Gerd Juchem, Martin Orban, Sven Peterss, Christian Hagl, Dominik Joskowiak

**Affiliations:** 1 Department of Cardiac Surgery, LMU University Hospital, Marchioninistrasse 15, 81377 Munich, Germany; germanherz@gmx.de (G.H.);; 2 Department of Political Science and Economics, Rowan University, Glassboro, NJ 08028, USA; 3 Department of Cardiology, LMU University Hospital, 81377 Munich, Germany; 4 German Center for Cardiovascular Research (DZHK), Partner Site Munich Heart Alliance, 81377 Munich, Germany; 5 Department of Anaesthesiology, LMU University Hospital, 81377 Munich, Germany

**Keywords:** postcardiotomy cardiogenic shock, extracorporeal life support, cardiocirculatory failure, mechanical circulatory support, health-related quality of life

## Abstract

**Background:** Extracorporeal life support (ECLS) therapy for refractory postcardiotomy cardiogenic shock (rPCS) is associated with high early mortality rates. This study aimed to identify negative predictors of mid-term survival and to assess health-related quality of life (HRQoL) and recovery of the survivors. **Methods:** Between 2017 and 2020, 142 consecutive patients received ECLS therapy following cardiac surgery. The median age was 66.0 [57.0–73.0] years, 67.6% were male and the median EuroSCORE II was 10.5% [4.2–21.3]. In 48 patients, HRQoL was examined using the 36-Item Short Form Survey (SF-36) and the modified Rankin-Scale (mRS) at a median follow-up time of 2.2 [1.9–3.2] years. **Results:** Estimated survival rates at 3, 12, 24 and 36 months were 47%, 46%, 43% and 43% (SE: 4%). Multivariable Cox Proportional Hazard regression analysis revealed preoperative EuroSCORE II (*p* = 0.013), impaired renal function (*p* = 0.010), cardiopulmonary bypass duration (*p* = 0.015) and pre-ECLS lactate levels (*p* = 0.004) as independent predictors of mid-term mortality. At the time of follow-up, 83.3% of the survivors were free of moderate to severe disability (mRS < 3). SF-36 analysis showed a physical component summary of 45.5 ± 10.2 and a mental component summary of 50.6 ± 12.5. **Conclusions:** Considering the disease to be treated, ECLS for rPCS is associated with acceptable mid-term survival, health-related quality of life and functional status. Preoperative EuroSCORE II, impaired renal function, cardiopulmonary bypass duration and lactate levels prior to ECLS implantation were identified as negative predictors and should be included in the decision-making process.

## 1. Introduction

Refractory postcardiotomy cardiogenic shock (rPCS) is defined as the inability to wean from cardiopulmonary bypass (CPB) or progressive hypoperfusion syndrome despite optimal medical treatment and volume resuscitation [1]. It affects approximately 0.2–3.7% of patients undergoing cardiac surgery and ECLS implantations related to cardiac surgery have clearly increased in the modern era [2,3,4,5,6]. While a reduction of ECLS-associated complications could be achieved with growing expertise, overall outcomes did not significantly improve [7]. Furthermore, early mortality remains persistently high with rates over 60%, even in high-volume centers [2,3,7,8]. Advanced age, female gender, preoperative neurologic events, prior cardiac surgery, aortic arch surgery and lactate levels above 6 mmol/L have been proven as independent risk factors associated with increased in-hospital mortality [3]. Whereas the early clinical course has been extensively described, evidence regarding outcomes beyond hospital discharge and patient recovery remains scarce [9,10,11]. The objective of this study was to identify independent pre-ECLS risk factors associated with mid-term mortality, and to evaluate the patient’s health-related quality of life (HRQoL) and physical disability after undergoing ECLS therapy for rPCS.

## 2. Materials and Methods

### 2.1. Study Population and Design

**Figure 1:**
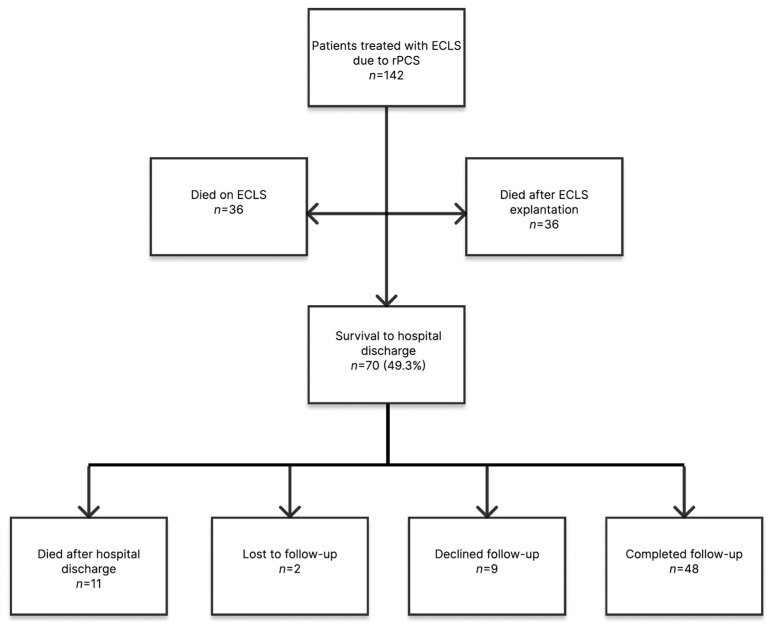
Patient cohort. ECLS, extracorporeal life support; rPCS, refractory postcardiotomy cardiogenic shock.

The presented study includes data of 142 consecutively treated patients with ECLS due to rPCS between January 2017 and December 2020 at our tertiary center. rPCS was defined as the inability to wean from CPB or progressive hemodynamic instability after CPB discontinuation despite optimal volume status and inotropic support. Exclusion criteria were age under 18 years, need of VV-ECMO due to isolated lung failure, ECLS for isolated right ventricular support after LVAD implantation and ECLS therapy initiated prior to or after more than 24 h since cardiac surgery because of other, non-surgery related causes of acute cardiac failure. The patient cohort is presented in Figure 1.

Patients who survived to hospital discharge were contacted by telephone for further assessment. Forty-eight patients (81.4% of survivors) underwent follow-up, which included a detailed evaluation of the patient’s overall morbidity and physical disability with the modified Rankin Scale (mRS), ranging between 0 (no disability) and 6 (death). HRQoL was obtained with the German version of the 36-Item Short Form Survey (SF-36), where 36 questions result in the mental component summary (MCS) and physical component summary (PCS), scoring from 0 (worse health) to 100 (better health). The component summaries consist of 8 subscales: physical functioning (PF), role physical (RP), bodily pain (BP), general health (GH), vitality (VT), social functioning (SF), role emotional (RE) and mental health (MH). A mean value of 50 ± 10 is considered within normal range. The primary endpoint was the mid-term outcome. Secondary endpoints were HRQoL and physical recovery at follow-up. Written consent was required from surviving patients. The study was approved by the institutional ethics committee of the Ludwig Maximilian University, Munich, Germany, on 25 May 2020 (Project No. 20-287).

### 2.2. ECLS Implantation Strategy and Technique

Our institutional policy encourages a liberal establishment of ECLS support and we do not impose absolute indications or contraindications. Patients are critically evaluated considering age, comorbidities, preoperative status, biventricular cardiac function, catecholamine doses, volume status, lactate levels and expected postoperative quality of life. The decision for ECLS implantation is made on an individual basis by the involved disciplines.

Central cannulation with temporary chest closure is the predominantly used technique for intraoperative ECLS initiation at our center. Percutaneous cannulation of the femoral vessels under fluoroscopy or transesophageal guidance is favored for secondary (postoperative) ECLS implantation. Hemodynamic assessment prior to and during ECLS support is routinely performed using a Swan–Ganz catheter. ECLS management and weaning are beyond the scope of this article and were recently published by our group [12].

### 2.3. Data Acquisition and Baseline Definitions

Patients meeting the inclusion criteria were selected from our institutional, Postcardiotomy Shock Registry database, which comprises retrospectively collected data from medical records and electronic hospital charts. This study focused on pre-ECLS parameters to offer support in the decision-making process as such. Baseline demographic characteristics, preexistent comorbidities, cardiac function, cardiovascular risk factors, hemodynamic status, blood gas analysis and surgical data were obtained. The underlying surgical procedures prior to ECLS implantation and the postoperative parameters are presented only in a descriptive manner. The extent of the performed surgical procedure is expressed by CPB duration and thus, the latter was included in the regression model. Impaired renal function (RF) before ECLS implantation was defined as either acute kidney injury with serum creatinine levels > 1.5 mg/dL with previously normal kidney parameters or chronic kidney disease, which was defined as serum creatinine > 1.5 mg/dL or GFR < 60 mL/min (using the CKD-EPI formula) for at least 3 months prior to surgery. The urgency status was classified according to the EuroSCORE II criteria [13]. Ischemic hepatitis included the elevation of alanine- or aspartate-aminotransferase > 1000 U/L. The vasoactive-inotropic score (VIS) represents the sum of all administered vasoactive and inotropic substances and was calculated with the doses used immediately before ECLS implantation [14]. 

### 2.4. Statistical Analysis

Categorial variables are presented as frequencies and percentages. Metric variables are presented as mean ± standard deviation or as medians and interquartile ranges if indications of non-normality were observed. Univariable Cox Proportional Hazard regression analysis was performed to analyze the impact of pre-ECLS parameters on mid-term outcomes. Parameters with *p*-values < 0.010 qualified for inclusion in the multivariable regression model and were chosen with consideration of clinical relevance. Survival probability was estimated using Kaplan–Meier survival analysis and the log-rank test was used to compare survival probabilities regarding pre-ECLS lactate levels and preoperative EuroSCORE II. A *p*-value of lower than 0.05 was considered statistically significant. Statistical analysis was performed with the statistical program R, 4.2.0.

## 3. Results

Between January 2017 and December 2020, 4868 patients underwent cardiac surgery with CPB. Thereof, 142 patients—2.9%—developed rPCS and received ECLS therapy within 24 h. The ECLS was established intraoperatively in 84.5% of the patients. The arterial cannula was placed in the aorta in 73.9% and the venous canula was inserted in the right atrium in 61.3% of the cases. Baseline characteristics and pre-ECLS parameters are presented in Table 1 and Table 2.

### 3.1. Early Outcome

Survival to hospital discharge was 49.3% (Figure 1). The median duration of ECLS therapy was 3.7 [2.1–5.5] days with a 72 h weaning success rate of 58.5%. Median ICU and hospital stay were 12.0 [5.0–23.0] and 16.5 [5.8–29.0] days, respectively. Concomitant left ventricular unloading with an Impella^®^, Abiomed, Danvers, MA, USA or with an intraaortic balloon counterpulsation was performed in 3.5% and 16.2% of the patients. The ECLS weaning via VV-ECMO was necessary in 5.6% due to prolonged pulmonary failure after recovery of cardiac function. The medium duration of invasive ventilation was 8.0 [4.0–19.3] days and 24.6% underwent tracheostomy. Adverse events after ECLS implantation were the following in decreasing frequency: new onset of renal replacement therapy (60.7%), re-exploration for bleeding (42.3%), new onset of atrial fibrillation (39.4%), ischemic hepatitis (28.2%), sepsis (27.5%), intestinal ischemia confirmed by abdominal CT scan (7.0%) and neurologic complication confirmed by cerebral CT scan (7.0%). Pump thrombosis requiring an exchange of the complete ECLS system occurred in 12.7%, while a solitary exchange of the oxygenator was required in 5.6% of the patients. Revision cardiac surgery was performed in 8.5% and percutaneous coronary intervention was necessary in 14.8% of the patients. Five patients (3.5%) received durable mechanical circulatory support. Two patients underwent paracorporeal BIVAD implantation (Berlin Heart EXCOR^®^, Berlin Heart, Berlin, Germany)—both died during the index hospitalization. A LVAD (HeartMate III, Abbott, Chicago, IL, USA) was implanted in three patients, from which two patients survived to hospital discharge.

### 3.2. Mid-Term Outcome

Cumulative survival was 41.5% (*n* = 59) during the observation period of 2.2 [1.9–3.2] years. Multiorgan failure and sepsis were the leading cause of death in 65% of the deceased patients. Multivariable regression analysis identified preoperative EuroSCORE II (*p* = 0.013), impaired RF (*p* = 0.010), CPB duration (*p* = 0.015) and pre-ECLS lactate levels (*p* = 0.004) as independent risk factors of mid-term mortality (Table 3). 

**Figure 2:**
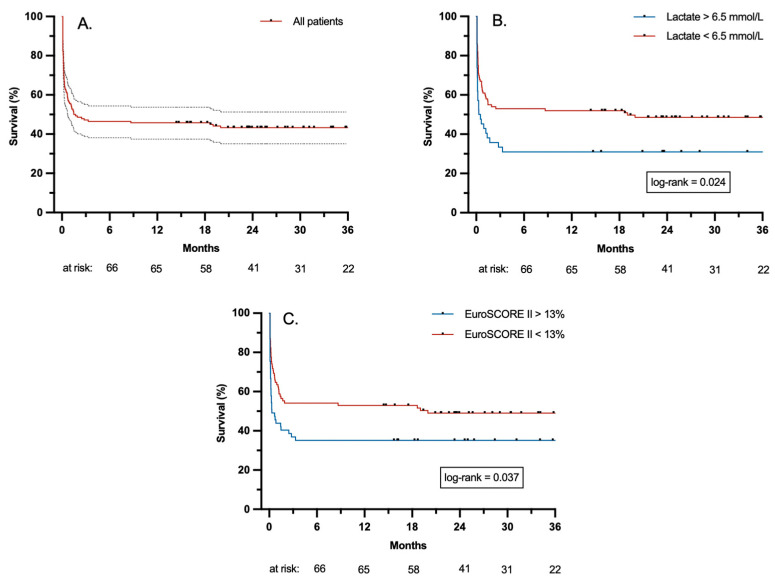
Estimated survival probabilities: (**A**) Kaplan–Meier curve of all 142 patients with 95% confidence interval. (**B**) Kaplan–Meier curves of patient groups divided by lactate levels. (**C**) Kaplan–Meier curves of patient groups divided by the EuroSCORE II.

**Figure 3:**
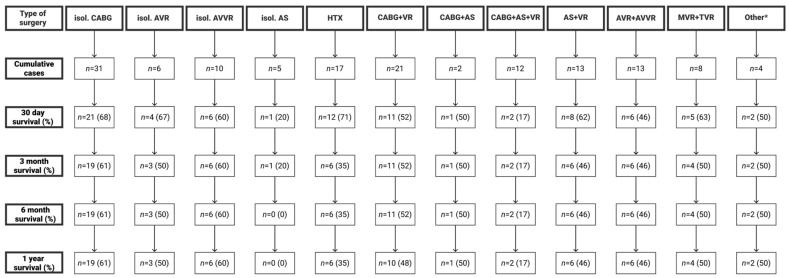
Outcome regarding surgical procedures: * 1 pulmonary thrombectomy, 1 ventricular septal defect closure, 2 pericardectomy; isol., isolated; CABG, coronary artery bypass graft; AVR, aortic valve replacement/repair; AVVR, atrioventricular valve replacement/repair; AS, aortic surgery; HTX, heart transplantation; VR, valve replacement; MVR, mitral valve replacement/repair; TVR, tricuspid valve replacement/repair.

The estimated survival probabilities are presented in Figure 2. Survival at 3, 12, 24 and 36 months were 47%, 46%, 43% and 43% (SE 4%), respectively. The log-rank test showed significantly reduced mid-term survival in patients with pre-ECLS lactate levels > 6.5 mmol/L (*p* = 0.024) and a EuroSCORE II > 13% (*p* = 0.037). Survival rates regarding the surgical procedures are summarized in Figure 3. 

### 3.3. Patient Status at Follow-Up

Forty-eight patients—81.4% of the survivors—consented to follow-up. Follow-up was unavailable in 11 patients. Nine patients declined further participation and two patients were lost to follow-up (Figure 1). The median age was 68 [60.3–73.0] years. Nineteen patients (39.6%) were of working-age (≤65 years). Thereof, 42.1% were employed and 42.1% were retired early prior the index event. At the time of follow-up, 21.1% were employed and 47.4% were retired. Twenty-nine patients (60.4%) were >65 years, from which 89.7% were retired and 10.3% were able to work part-time. 

Out of all patients, 83.3% were NYHA I-II and 16.7% were NYHA III. While 54.2% felt somehow impaired in everyday life activities, objective measurements showed a higher grade of self-sufficiency. Eight patients (16.7%)—seven were older than 65 years—suffered from moderate to severe physical disability (mRS ≥ 3). Six patients (12.5%)—four were older than 65 years—required care. After hospital discharge, 2.1% suffered from a stroke and 2.1% of the patients required new onset of renal replacement therapy. None of them underwent cardiac reoperation or groin surgery. Further cardiac interventions after discharge were necessary in 8.3% of the cases (coronary intervention: three patients, cardiac ablation: one patient). An implantable cardioverter defibrillator was implanted in 6.3% of the patients (primary prophylaxis: two patients; secondary prophylaxis after cardiopulmonary resuscitation due to ventricular fibrillation: one patient). Results of the SF-36 analysis are presented in Table 4. 

## 4. Discussion

Temporary mechanical circulatory support represents the only therapeutic option for prolonged cardiac reperfusion and circulatory stabilization in cases of rPCS. These patients often face a complicated postoperative course, requiring prolonged treatment concepts with significant early mortality [3]. Postcardiotomy ECLS support remains a complex and resourceful therapy for marginal patients and its benefit beyond hospital discharge is insufficiently described. The presented study shows the following:

(1) ECLS therapy for rPCS was associated with high mortality within the first 3 months, but survival rates stabilized thereafter.
(2) Mid-term prognosis was significantly compromised in patients with preoperatively impaired RF, higher EuroSCORE II and pre-ECLS lactate levels as well as longer CPB duration. Furthermore, patients with pre-ECLS lactate levels > 6.5 mmol/L and an EuroSCORE II > 13% had worse outcomes.
(3) HRQoL 2 years after ECLS therapy was decent. In patients with reduced HRQoL, the impairment was mostly attributed to physical restrictions (low PCS scores). Overall recovery was positive and only a small proportion (mostly affecting patients older than 65 years at the time of follow up) suffered from moderate to severe physical disability.

The mid-term survival following rPCS was acceptable in the presented study and generally higher compared to previously published data [2,3,15]. However, the trend over the subsequent years remained similar. In two multicenter studies, each including more than 500 patients, survival to hospital discharge and 1-year survival were only 25% and 17% [2] and 36% and 33% [3], respectively. A single-center analysis, including 360 patients, also revealed a reduced survival rate of 30% at hospital discharge, followed by a 1-year survival of 26% [15]. The differences in outcome are most probably a consequence of institutional-specific treatment approaches and inhomogeneous patient cohorts. Our institutional policy includes an early and predominantly intraoperative initiation of ECLS support, which may have impacted the better outcome. In our patient cohort, ECLS support was established intraoperatively in 84.5% of the cases and at earlier signs of hypoperfusion (median lactate levels of 4.7 mmol/L), whereas several studies with worse outcomes reported on higher initial lactate levels (average values > 7 mmol/L) and less common intraoperative ECLS establishment, namely in 42–70% of the cases [2,3,4,5,16]. In terms of the estimated preoperative morbidity, the median EuroSCORE II was lower in our patient cohort than in two multicenter studies, which could have affected the higher survival rates as well [2,3]. The average logistic EuroSCORE in the study by Rastan et al. was 22% [2] and Biancari et al. reported on an average EuroSCORE II of 16% [3]. 

Both factors, pre-ECLS lactate levels and preoperative EuroSCORE II, were identified as independent negative predictors. Our results are consistent with previously published studies [2,5,6,15,16]. Pre-ECLS lactate levels > 6.5 mmol/L were significantly associated with decreased mid-term survival. This correlates with the study of Biancari et al., who described a significantly increased in-hospital mortality at lactate levels > 6 mmol/L [3]. Schrutka et al. examined the discriminatory power of various scores for outcome prediction in patients with rPCS and found that the EuroSCORE II was a useful prediction model for long-term mortality [6]. 

Impaired RF was a further negative predictor of mid-term outcome. Hedley et al. described the correlation between preoperative renal failure, regardless of the stage, and outcome after cardiac surgery per se. The authors concluded that impaired RF not only increased the risk of postoperative complications and longer hospital stay, but also increased the risk of operative mortality [17]. Rastan et al. identified impaired RF as an independent risk factor of in-hospital mortality in patients with rPCS as well [2].

Prolonged time on cardiopulmonary bypass, often a consequence of complex surgical procedures or intraoperative technical difficulties, significantly impaired outcomes. It seems to be even more relevant in cases of rPCS. The result is consistent with a smaller study by Mashiko et al., in which CPB duration > 270 min significantly led to higher rates of in-hospital mortality in patients with rPCS [18].

Because of the increased vulnerability, intraoperative screening for signs of hypoperfusion and early ECLS implantation, especially in patients with prolonged CPB duration, is generally preferred at our institution.

At the time of follow up, recovery was predominantly positive, especially in patients younger than 65 years. Nonetheless, recovery did not result in the ability to work in most working-age patients. While age was not identified as a risk factor of mortality, it seems to be relevant for recovery. A small proportion of patients suffered from impairing physical restrictions and were dependent on care, mostly affecting patients older than 65 years. Otherwise, the majority (>80%) showed little symptoms of heart failure (NYHA I-II) and remained free of moderate to severe functional impairment (mRS ≥ 3). Nevertheless, half of the patients, regardless of age, felt subjectively impaired in everyday life activities. Papadopoulos et al. reported on 77% of the surviving patients being NYHA I–II five years after rPCS and a readmission rate of 56% due to cardiac decompensation or pneumonia [15].

HRQoL was decent in our patient cohort. On average, both component summary scales and all subscales were within or above normal range. In patients with impaired HRQoL, reductions in the subscales BP, RP and VT were crucial. Two smaller studies support our findings [9,11]. Shao et al. examined patients suffering from rPCS and compared patients who received ECLS therapy with patients who underwent cardiac surgery and did not receive ECLS therapy. While outcomes beyond 30 days were comparable between groups, HRQoL (SF-36 subscales GH and VT) was significantly lower in patients undergoing ECLS therapy. However, the authors concluded that HRQoL improved over time and stabilized after the third year following rPCS, which underlines the prolonged process of recovery [11]. Norkiene et al. examined 15 patients after a median follow-up time of 5.8 years and obtained similar PCS and MCS scores (PCS: 46.1 ± 7 and MCS: 47.1 ± 8) [9]. The authors additionally identified post-traumatic stress disorder in one third of the patients [9]. HRQoL in our patient cohort was also comparable with patients after extracorporeal cardiopulmonary resuscitation and ECLS support due to refractory cardiogenic shock without prior cardiac surgery [19,20,21]. 

In conclusion, considering the severity of rPCS, overall outcomes in terms of functional status and HRQoL can be interpreted as satisfactory. Nevertheless, a small proportion of these patients suffered from relevant physical disabilities correlating with diminished quality of life. Thus, patients surviving rPCS should be enrolled in prolonged intensive rehabilitation programs for improved outcome beyond absolute survival.

## 5. Limitations

This is a retrospective single-center study with the inherent limitation of such an analysis. The observational design and relatively small sample size are limiting factors. The follow-up was performed only once after variable observation periods. It was unavailable in 11 patients and thus, potentially resulted in a sampling bias. Additionally, preoperative HRQoL and physical disability were not obtained, which could have influenced outcome at follow-up as well. Nonetheless, this study is based on a realistic patient collective and provides valuable information for the decision-making process. Further studies with larger sample sizes are necessary to validate our findings.

## 6. Conclusions

The presented study indicates that the use of ECLS is not only a feasible therapeutic option in patients suffering from rPCS, but also leads to an acceptable mid-term outcome if the patient overcomes the first three months after the index event. Decent recovery and perceived health-related quality of life can be achieved in survivors, especially in younger patients. The preoperative EuroSCORE II, impaired renal function, extent of surgery expressed by CPB duration and lactate levels potentially indicate a worse outcome and should be considered in the decision-making process. Furthermore, patients presenting signs of hypoperfusion after cardiac surgery should be critically evaluated for ECLS support at early stages. 

## Figures and Tables

**Table 1:** Patient characteristics and preoperative data.

Variable	All Patients *n* = 142, *n* (%)	Survivors *n* = 59,*n* (%)	Non-Survivors *n* = 83, *n* (%)	HR	*p*-Value
**Male**	96 (67.6)	39 (66.1)	57 (68.7)	0.98 (0.6–1.6)	0.935
Age (years)	66.0 (57.0–73.0)	66.0 (58.0–72.0)	67.0 (57.0–75.0)	1.01 (0.99–1.04)	0.262
BMI (kg/m^2^)	26.5 (23.7–30.6)	27.8 (24.3–31.7)	26.1 (23.1–29.5)	0.96 (0.91–1.00)	0.067
EuroSCORE II	10.5 (4.2–21.3)	9.2 (3.0–16.7)	11.6 (6.4–27.7)	1.03 (1.02–1.05)	***<0.001***
**CAD**	86 (60.6)	36 (61.0)	50 (60.2)	0.96 (0.62–1.49)	0.852
Triple-vessel CAD	59 (41.5)	24 (40.7)	35 (42.2)	0.98 (0.63–1.52)	0.930
ACS in the last 90 days	39 (27.5)	20 (33.9)	19 (22.9)	0.74 (0.44–1.23)	0.241
LM stenosis > 50%	26 (18.3)	11 (18.6)	15 (18.1)	1.03 (0.59–1.80)	0.921
**Cardiomyopathy**	45 (31.7)	21 (35.6)	24 (28.9)	0.72 (0.45–1.16)	0.179
Ischemic cardiomyopathy	27 (19.0)	13 (22.0)	14 (16.9)	0.74 (0.41–1.31)	0.296
Dilated cardiomyopathy	17 (12.0)	7 (11.9)	10 (12.0)	0.89 (0.46–1.73)	0.729
Other cardiomyopathy ^*^	1 (0.7)	1 (1.7)	0 (0)		
**Cardiovascular risk factors**					
Arterial hypertension	111 (78.2)	44 (74.6)	67 (80.7)	1.29 (0.75–2.23)	0.361
Hyperlipoproteinemia	95 (66.9)	40 (67.8)	55 (66.3)	0.97 (0.62–1.53)	0.895
Diabetes mellitus ^§^	38 (26.8)	14 (23.7)	24 (28.9)	1.15 (0.72–1.85)	0.563
Smoking	60 (42.3)	27 (45.8)	33 (39.8)	0.84 (0.54–1.30)	0.426
**Preexistent comorbidities**					
COPD ^&^	11 (7.7)	3 (5.1)	8 (9.6)	1.51 (0.73–3.13)	0.271
Impaired RF	73 (51.4)	25 (42.4)	48 (57.8)	1.79 (1.15–2.77)	***0.009***
RRT ^†^	7 (4.9)	4 (6.8)	3 (3.6)	0.63 (0.20–1.98)	0.426
Stroke	31 (21.8)	15 (25.4)	16 (19.3)	0.87 (0.51–1.50)	0.621
Peripheral artery disease ^#^	19 (13.4)	8 (13.6)	11 (13.3)	1.15 (0.61–2.17)	0.664
Pulmonary hypertension	52 (36.6)	23 (39.0)	29 (34.9)	0.88 (0.56–1.39)	0.584
Atrial fibrillation	52 (36.6)	19 (32.2)	33 (39.8)	1.21 (0.78–1.88)	0.392
Endocarditis	18 (12.7)	4 (6.8)	14 (16.9)	2.13 (1.20–3.81)	***0.010***
**Echocardiography**					
LVEF (%)	50.0 (30.0–60.0)	50.0 (29.5–60.0)	49.0 (30.0–60.0)	1.00 (0.99–1.02)	0.582
Normal LVEF	60 (42.3)	27 (45.8)	33 (39.8)	0.87 (0.56–1.36)	0.545
Mildly impaired LVEF	20 (14.1)	7 (11.9)	13 (15.7)	1.38 (0.76–2.49)	0.291
Moderately impaired LVEF	18 (12.7)	6 (10.2)	12 (14.5)	1.49 (0.80–2.74)	0.206
Severely impaired LVEF	44 (31.0)	19 (32.2)	25 (30.1)	0.81 (0.50–1.29)	0.367
TAPSE < 16 mm	49 (34.5)	15 (25.4)	34 (41.0)	1.40 (0.90–2.17)	0.135
Severe AV regurgitation	14 (9.9)	4 (6.8)	10 (12.0)	2.02 (1.04–3.91)	***0.039***
Severe MV regurgitation	33 (23.2)	18 (30.5)	15 (18.1)	0.63 (0.36–1.10)	0.106
Severe TV regurgitation	23 (16.2)	8 (13.6)	15 (18.1)	1.42 (0.81–2.50)	0.218
Invasive ventilation	20 (14.1)	8 (13.6)	12 (14.5)	1.18 (0.64–2.17)	0.599

HR, hazard ratio; BMI, body mass index; CAD, coronary artery disease; ACS, acute coronary syndrome; LM, left main coronary artery; COPD, chronic obstructive pulmonary disease; RF, renal function; RRT, renal replacement therapy; LVEF, left ventricular ejection fraction; TAPSE, tricuspid annular plane systolic excursion; AV, aortic valve; MV, mitral valve; TV, tricuspid valve; ^*^ suspected right ventricular arrhythmogenic cardiomyopathy; ^§^ diabetes mellitus with dependence of insulin or oral antidiabetic agents; ^&^ COPD of all stages; ^†^ preexistent permanent RRT and RRT due to acute kidney injury before surgery; ^#^ peripheral artery disease of all stages.

**Table 2:** Operative and pre-ECLS data.

Variable	All Patients *n* = 142, *n* (%)	Survivors *n* = 59, *n* (%)	Non-Survivors *n* = 83, *n* (%)	HR	*p*-Value
**Cardiac reoperation**	50 (35.2)	16 (27.1)	34 (41.0)	1.44 (0.93–2.24)	0.104
Elective surgery	46 (32.4)	17 (28.8)	29 (34.9)	1.07 (0.68–1.69)	0.755
Urgent surgery	65 (45.8)	28 (47.5)	37 (44.6)	0.95 (0.61–1.46)	0.811
Emergency surgery	31 (21.8)	14 (23.7)	17 (20.5)	0.98 (0.58–1.67)	0.945
Combined surgery	71 (50.0)	29 (49.2)	42 (50.6)	1.20 (0.78–1.85)	0.399
**CPB times**					
CPB duration (min)	215.0 (145.5–283.5)	204.0 (134.0–280.0)	223.5 (160.8–286.8)	1.00 (1.00–1.01)	***0.028***
Cross clamp time (min)	118.0 (78.0–165.3)	107.0 (70.0–157.0)	131.0 (85.5–171.0)	1.00 (1.00–101)	***0.021***
Circulatory arrest	25 (17.6)	9 (15.3)	16 (19.3)	1.39 (0.80–2.40)	0.240
**Pre-ECLS data**					
CPR	25 (17.6)	10 (16.9)	15 (18.1)	1.11 (0.64–1.95)	0.710
pH	7.36 (7.31–7.40)	7.36 (7.32–7.40)	7.36 (7.29–7.41)	0.73 (0.05–10.13)	0.814
Lactate (mmol/L)	4.7 (3.1–6.9)	4.3 (2.8–6.4)	5.0 (3.2–7.2)	1.07 (1.01–1.12)	***0.014***
PEEP (cmH_2_O)	10.0 (8.0–12.0)	10.0 (10.0–12.0)	10.0 (8.0–12.0)	0.94 (0.87–1.02)	0.161
Pinsp (cmH_2_O)	20.0 (18.0–25.0)	20.0 (18.0–24.0)	20.0 (18.0–27.0)	1.01 (0.97–1.04)	0.731
VIS	43.2 (30.4–60.2)	43.9 (31.3–59.4)	41.0 (29.2–62.8)	1.00 (0.99–1.01)	0.904

HR, hazard ratio; CPB, cardiopulmonary bypass; CPR, cardiopulmonary resuscitation; PEEP, positive end expiratory pressure; Pinsp, peak inspiratory pressure; VIS, vasoactive-inotropic score.

**Table 3:** Multivariable regression model.

Variable	Univariable Regression		Multivariable Regression	
	HR	*p*-Value	HR	*p*-Value
BMI (kg/m^2^)	0.96 (0.91–1.00)	0.067	0.97 (0.92–1.01)	0.119
EuroSCORE II	1.03 (1.02–1.05)	<0.001	1.02 (1.00–1.04)	***0.013***
Impaired RF	1.79 (1.15–2.77)	0.009	1.93 (1.17–3.20)	***0.010***
Severe AV regurgitation	2.02 (1.04–3.91)	0.039	1.34 (0.64–2.81)	0.432
Endocarditis	2.13 (1.20–3.81)	0.010	1.04 (0.51–2.12)	0.920
CPB duration (min)	1.00 (1.00–1.01)	0.028	1.003 (1.00–1.01)	***0.015***
Lactate (mmol/L)	1.07 (1.01–1.12)	0.014	1.08 (1.02–1.14)	***0.00*** ***4***

HR, hazard ratio; BMI, body mass index; RF, renal function; AV, aortic valve; CPB, cardiopulmonary bypass.

**Table 4:** Health-related quality of life.

36-Item Short Form Survey	Mean ± SD	Below Normal Range *n* (%)
Physical component summary	45.5 ± 10.2	13 (27.1)
Mental component summary	50.6 ± 12.5	7 (14.6)
Physical functioning	71.9 ± 27.3	6 (12.5)
Role physical	71.9 ± 37.1	9 (18.8)
Bodily pain	71.9 ± 32.5	9 (18.8)
General health	67.2 ± 22.6	6 (12.5)
Vitality	60.9 ± 25.2	8 (16.7)
Social functioning	80.2 ± 26.1	6 (12.5)
Role emotional	88.9 ± 29.4	5 (10.4)
Mental health	76.0 ± 19.1	2 (4.2)

## Data Availability

The data underlying this article are available in the article.

## Abbreviations

BIVAD	biventricular assist device
BP	bodily pain
CPB	cardiopulmonary bypass
ECLS	extracorporeal life support
GH	general health
HRQoL	health-related quality of life
ICU	intensive care unit
LVAD	left ventricular assist device
MCS	mental component summary
MH	mental health
mRS	modified Rankin Scale
PCS	physical component summary
PF	physical functioning
RE	role emotional
RF	renal function
RP	role physical
rPCS	refractory postcardiotomy cardiogenic shock
SF	social functioning
SF-36	36-Item Short Form Survey
VIS	vasoactive-inotropic score
VT	vitality
VV-ECMO	veno-venous extracorporeal membrane oxygenation
